# The Aquatic Communities Inhabiting Internodes of Two Sympatric Bamboos in Argentinean Subtropical Forest

**DOI:** 10.1673/031.013.9301

**Published:** 2013-09-30

**Authors:** Raúl E. Campos

**Affiliations:** 1 Instituto de Limnología “Dr. Raúl A. Ringuelet”, Universidad Nacional de La Plata — CONICET, CC 712 (1900) La Plata, Buenos Aires, Argentina; 2 Consejo Nacional de Investigaciones Científicas y Técnicas (CONICET)

**Keywords:** community, *Guadua chacoensis*, *Guadua trinii*, insect association, mosquito, phytotelmata, predators

## Abstract

In order to determine if phytotelmata in sympatric bamboos of the genus *Guadua* might be colonized by different types of arthropods and contain communities of different complexities, the following objectives were formulated: (1) to analyze the structure and species richness of the aquatic macroinvertebrate communities, (2) to comparatively analyze co-occurrences; and (3) to identify the main predators. Field studies were conducted in a subtropical forest in Argentina, where 80 water-filled bamboo internodes of *Guadua chacoensis* (Rojas Acosta) Londoño and Peterson (Poales: Poaceae) and *G. trinii* (Nees) Nees and Rupr. were sampled. Morphological measurements indicated that *G. chacoensis* held more fluid than *G. trinii*. The communities differed between *Guadua* species, but many macroinvertebrate species used both bamboo species. The phytotelmata were mainly colonized by Diptera of the families Culicidae and Ceratopogonidae.

## Introduction

Phytotelmata (gr. *phyton* = plant, *telma* = pond) occur in a large number of terrestrial plants that hold water in places such as stems, sheathing leaves, and flowers (Frank and Lounibos 1983). Extensive lists of types of phytotelmata have been presented by Fish (1983) and Machado-Allison et al. (1985), although only a few types have been extensively studied. Bromeliads, pitcher plants, treeholes, and bamboo internodes have been among the most studied phytotelmata (e.g., Kitching 1971; Istock et al. 1975; Louton et al. 1996; Frank and Lounibos 2009) because their fauna may include dipteran vectors of diseases (Anosike et al. 2007), and because they are suitable for studies on ecological systems (e.g., Yee 2007). Treeholes and bamboo were termed as dendrotelmata (gr. *déndron* = tree, *telma* = pond) due to characteristics that differentiate them from other phytotelmata. The main feature that distinguishes them is that the pool is formed in the trunk or woody stem (Lozovei 1998), in contrast with the other plants that are herbaceous. Arguably, bamboos constitute the simplest phytotelmata from the viewpoint of their architecture. Each internode has the shape of a hollow cylinder, with smooth and vertical walls, and both ends are closed. The pool is formed when the internode is filled with rainwater (Kovac and Streit 1996) or the exudates of the bamboo (Lozovei 1998). Rainwater falls within the internodes either when the top is cut, which may give rise to two types of bamboo-stump pools, open, or semi-closed (Sunahara and Mogi 1997), or when the culm is injured by an insect borer such a chrysomelid (Macdonald 1962) or curculionid (Kovac and Azarae 1994) beetle. It must be emphasized that closed internodes accumulate fluids but become phytotelmata only when drilled or injured, allowing female insects to enter and lay eggs (Lozovei 1998).

Studies of the effect of habitat size in treeholes and bamboo stumps showed that the size of the opening determines the rate of entry of leaf litter, which increases with phytotelm capacity (Sota 1996). In turn, an increase in the capacity of the habitat should lead to increases in biomass and the number of metazoan inhabitants. Furthermore, it was demonstrated that the stability of phytotelmata has an additional effect on community structure, due to the effect of stability of the aquatic environment. Stability is influenced by hole depth (Sota et al. 1994), as small deep holes are more stable than large shallow holes (Sota 1996). Therefore, the number of individuals and species composition varies with the capacity of the treehole. Thus, mosquito species whose egg stages are not resistant to drought colonize more stable treeholes than species with drought-resistant eggs (Bradshaw and Holzapfel 1983). The stability of the aquatic micro-environment in bamboo internodes could be greater than the stability in treeholes or bamboo stumps because the opening area where rainwater enters is generally small and lateral on internodes, as opposed to stumps that have a larger opening area facing up, like those of many treeholes. On the other hand, water-holding capacity of internodes will not depend on their length, but on the height where the side hole is.

Many studies have been conducted on bamboo phytotelmata, addressing different aspects of the communities, mainly communities from stumps. For example, the species richness in an altitudinal gradient (Sota and Mogi 1996) was studied, as well as the seasonal occurrence and distribution patterns of mosquitoes in bamboo groves (Sunahara and Mogi 1997) and the variability of intra and interspecific competition of mosquitoes in stumps (Sunahara and Mogi 2002). The communities of aquatic macrofauna of internodes were studied in a lowland tropical forest (Louton et al. 1996), but there are no studies comparing the communities living in internodes of sympatric bamboo species. From observations made during a study on the diversity of Culicidae and Ceratopogonidae that inhabit phytotelmata in the subtropical region of Argentina (Campos et al. 2011), it was found that two species of the bamboo genus *Guadua* had differences in morphology and presumably are able to accommodate different detritus. Therefore, it was speculated that the species richness and community structure of macroinvertebrates could be different. To substantiate this hypothesis, the following objectives were formulated: (1) to compare the structure and species richness of the aquatic macroinvertebrate communities in two sympatric species of bamboo; (2) to compare the co-occurrence of aquatic macroinvertebrates between the two bamboos species, and (3) to identify the main predators in the aquatic communities of both bamboos.

## Materials and Methods

### Study area and bamboo species

Field studies were conducted from 15–19 March 2007 in Iguazú National Park (25° 39′ S, 54° 18′ W), located in the northwest of Misiones Province, Argentina, by the Iguazú River on the border of Brazil. The park is situated in the Paranaense forest ecoregion (Dinerstein et al. 1995), which has a topography and drainage pattern dominated by a basaltic plateau, reaching altitudes of 700 m a.s.l. The dominant vegetation corresponds to the semi-deciduous forest, and the climate is subtropical, characterized by dry winters and wet summers, with an annual rainfall of 1,500– 2,000 mm. The mean temperature varies from 16–22° C (APN 2009).

Iguazú National Park holds two kinds of bamboo (Poaceae), woody (Bambuseae) andherbaceous (Olyreae). *Guadua* Kunth, *Merostachys* Sprengel, and *Chusquea* Kunth are the three genera of woody native bamboo (Probambu 2011), but only *Guadua* and *Merostachys* species, whose stems are hollow, produce phytotelmata. In this study, *Guadua* (Rojas Acosta) Londoño and Peterson (Poales: Poaceae) and *G. trinii* (Nees) Nees and Rupr. were sampled, but *Merostachys clausenii* Munro was not sampled, because at the end of 2006 this species bloomed (it occurs approximately every 30 years, followed by a massive die-off, leaving large areas deforested). Both species of *Guadua* grow in the forest, mainly in low places near streams. *Guadua chacoensis* is dominant and grows in clumps occupying big areas, while *G. trinii* grows in small clumps scattered in the forest. However, both bamboos are often found together.

### Sampling

The preferred method for sampling internodes of bamboo is to cut the internode into sections to remove all contents. However, in our study, a different method was used because the bamboos were in an area of biodiversity conservation. For this reason, only the aquatic macroinvertebrates were considered in this research, and the terrestrial macroinvertebrates that use the internodes as refuge were not considered.

The study was carried out in early fall, after the summer rainfall. Thirty-nine water-filled bamboo internodes of *G. chacoensis* and 41 of *G. trinii* were sampled. Standing water containing macroinvertebrate inhabitants was extracted with a pooter (aspirator) attached to a lift pump, and the volume of water was measured. Because the diameters of the natural holes of the internodes were in some cases smaller than the suction hose, new larger (2.5 cm) holes were made with a drill, just above the level of the original holes. Internodes were washed and re-extracted several successive times with clean tap water until no macroinvertebrates or sediments were observed in the extracted water. No bamboo stumps were considered for this study.

All macroinvertebrates were killed in the field and preserved in 80% commercial ethyl alcohol, except some immature forms, which were carried alive to the laboratory in individual plastic tubes to be reared and identified. After removal of all macroinvertebrates, the fluid extracted (original siphoning plus washes) from the internodes was filtered, and the sediment was dried at 60° C for 48 hr, and weighed on a microbalance accurate to 0.01 mg.

The inner diameter; depth (*de*) (measured from bottom to level of the hole); wall thickness; height; and total length (*l*) of each bamboo internode and size of holes in the wall were measured. The percentage of the length of the internodes able to hold water was calculated as 100*de* / *l*. Capacity (c) of each internode was determined as *πi^2^de* / 4 (Sota and Mogi 1996). Water and air temperature were measured before water extraction with a digital thermometer equipped with a probe, and pH was measured with a portable pH meter after extraction.

Taxonomic resolution was attempted to species level, and for this reason some late instar larvae and pupae of insects recognized as different morphospecies were grown to adulthood. Culicidae were identified by the author using the keys of Darsie (1985), Ceratopogonidae were identified by a specialist, and the other taxa were identified to order or family using the keys of Stehr (1987, 1991).

### Data analysis

Significant variation in the water volume, pH, and sediment among *Guadua* species was tested with Student (*t*) and Mann-Whitney tests (*U*).

Principal component analysis was used to study physical, chemical, and morphological variations of bamboo phytotelmata (Ter Braak and Smilauer 1998). All data were standardized previous to analysis (Ter Braak 1986). A complementary non-parametric Mann-Whitney test was used to estimate differences and their significance between variables.

To graph the frequency distribution of the size of the holes in the bamboo internodes, they were categorized into seven classes: 0–5, 5.1– 10, 10.1–15, 15.1–20, 20.1–25, 25.1–30, and 30.1–35 mm.

The diversity of macroinvertebrates from each *Guadua* bamboo was measured by Shannon-Wiener index (*H*') (Magurran 1988), and differences between *H*' indexes calculated for each *Guadua* species were tested with Student's *t*-test (Zar 1996). The evenness (*E*) of the numbers of taxa in each sample was estimated using Pielou's index (Pielou 1975). The dominance of taxa in both *Guadua* species was studied using the Berger-Parker index (Magurran 1988), which was calculated for each internode. Differences in *d* indexes among *Guadua* species were tested by Student' s *t*-test (Zar 1996).

To examine interespecific associations among macroinvertebrates within each bamboo species, the C_8_ coefficient, which varies from +1 to -1 (Hulbert 1969), was used, and significance association was tested by χ^2^ corrected for continuity.

**Table 1.**
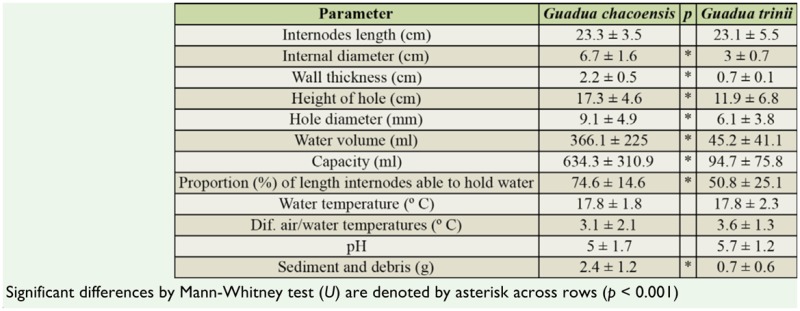
Mean ± standard deviation of morphometric data from 81 internodes of two bamboo phytotelmata from subtropical forest, Misiones Province, Argentina.

Multiple regression analyses were used to study the effect of abiotic parameters of each bamboo phytotelmata on all macroinvertebrates, and on mosquitoes in particular. Height of the internodes, capacity, size of inlet hole, water volume held, pH, and debris were considered as independent variables, and number of individuals and number of taxa were the response variables. Dependent variables were transformed as square root (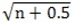), and independent variables as log10, before analysis to accommodate assumptions of normality and homogeneity of variance (Zar 1996). Collinearity was tested by the variance inflation factor (*VIF*), and correlation between the residuals by the Durbin-Watson statistic (*D*) (Santos and Luque 1996). Variables that showed collinearity were excluded from analysis (Phillipi 1993). Complementary correlation coefficients of Spearman (*r_s_*) were calculated between number of mosquitoes per genera and pH for the two bamboos.

Significant variation in the abundances of macroinvertebrates inhabiting internodes among holes of different sizes was tested with a one way-analysis of variance. If significant variation was detected, *a posteriori* comparisons were performed to identify significant differences between groups.

The proportion of both *Guadua* species containing immatures of *Toxorhynchites* species in internodes with a hole entry less and greater than 10 mm of diameter was compared by *G_adj_*. test.

### Results

#### Bamboo internodes as micro-habitats

Eighty bamboo internodes were measured, 39 of which were from *G. chacoensis* and 41 from *G. trinii* (Table 1). The lengths of the internodes of both *Guadua* species were not significantly different (*U=* 1655; n = 39–42; *p* = 0.6). Internal diameter, wall thickness, capacity, water volume, sediment, and proportion of internode length able to hold water were significantly greater in *G. chacoensis* than in *G. trinii* (*U _Diameter_* = 2414; *U _Thickness_* = 2418; *U _Capacity_* = 2391; *U _Water_* =2380.5; *U _Sediment_* = 2130; *U _Proportion_* = 2044; n = 39–42; *p* < 0.001). The pH was acid in both bamboos but not was significantly different (*U* = 1401.5; n = 34–38; *p* = 0.071). In both bamboos, the small holes dominated, as 61.5% of the internodes of *G. trinii* had holes less than 5 mm, while 64.1% of *G. chacoensis* had holes ranging from 5.1–10 mm. However, the holes in *G. chacoensis* were located significantly higher than those in *G. trinii* (*U _Hole diameter_* = 1994.5; *U _Height of hole_* = 1990.5; n = 39–42; *p* < 0.001). Water temperature and difference between air and water temperatures in both *Guadua* species were not significantly different (*t*_*water temperature*_ = 0.0273; df = 79; *p* = 0.978; *U*_*Air/water temperature*_ = 1385.5; n = 39–42; *p* = 0.044).

**Figure 1.**
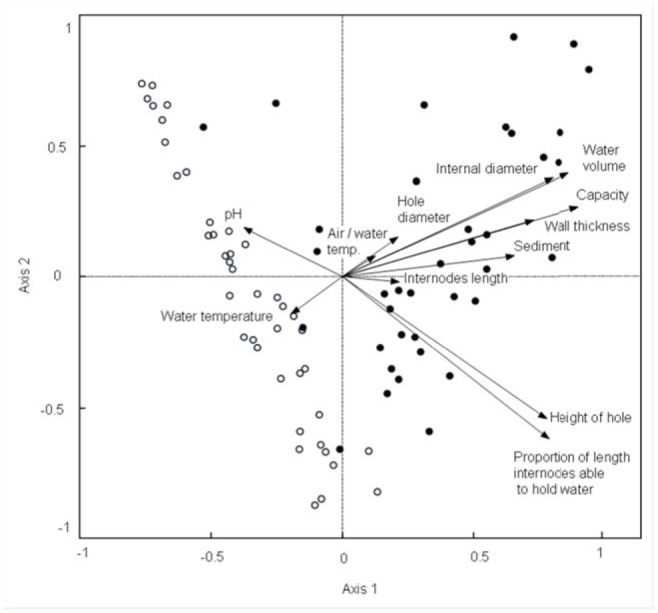
Relationship of physical, chemical, and morphological variables with the first two axes of principal component analysis. Black circles represent *Guadua chacoensis* and the white represent *G. trinii*. High quality figures are available online.

The first three axes of principal component analysis explained 87.5% of the total variance. The first, second, and third axes explained 62.6%, 18.2%, and 6.7% respectively, and are associated with the variables shown in Table 2. The principal component analysis ordination diagram (Figure 1) shows two clusters segregated along CC1. On negative axis 1, all except two *G. trinii* internodes are grouped, while on positive axis 1 the majority of *G. chacoensis* internodes are found, showing that they are bigger and hold more water and sediment than *G. trinii* internodes. All variables are positively correlated with axis 1 except for water temperature and pH. The capacity and the volume of water held in the internodes are the variables best correlated with axis 1 (*r*^2^ = 0.90 and 0.86 respectively). The pH of the water retained in both bamboos was acidic (Table 1), and the greater variability was observed in *G. chacoensis* (*C. V_G. chacoensis_* = 33.6%; *C.V _G. trinii_* = 22.3%). The principal component analysis diagram shows that the pH of *G. trinii* is more likely to be more acid than in *G. chacoensis*, however significant differences were not observed (Table 1).

**Table 2.**
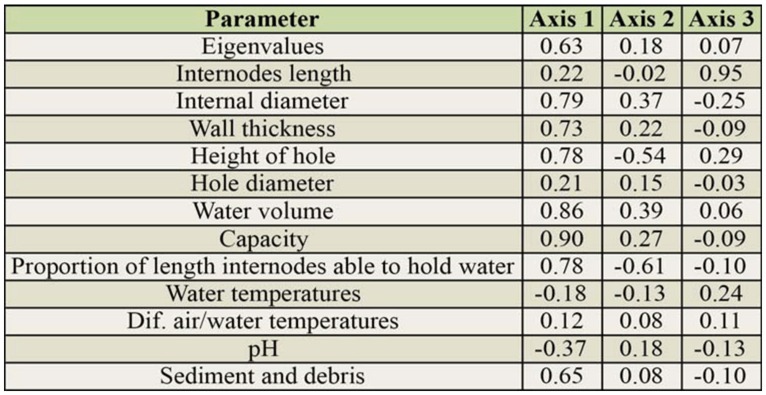
Eigenvalues and correlation coefficients (*r*^2^) of parameters associated with the axes obtained in principal component analysis.

#### Fauna communities

The number of individuals collected from *G. chacoensis* and *G. trinii* was 1,752 and 1,073 respectively, distributed by taxonomic group as shown in Table 3. The number of individuals per internode was higher and significantly different (*U* = 1921; *n* = 39, 41; *p* < 0.001) in *G. chacoensis* (Mean = 44.9; SD = 36.8) than in *G. trinii* (Mean = 26.2; SD = 32.1).

The total number of taxa identified was 18 from *G. chacoensis* and 20 from *G. trinii*; however, for analyses, 17 and 19 respectively were considered because larvae of 2 species of *Toxorhynchites* were indistinguishable (Table 3). Mean number of taxa by individual internode was 4.6 (± SD 1.4) and 4 (± SD 1.8) for *G. chacoensis* and *G. trinii* respectively, but they were not statistically different (*U* = 1766; *n* = 39, 41; *p* = 0.07). The maximum number of taxa found in an internode was 8 for both bamboo species.

**Table 3.**
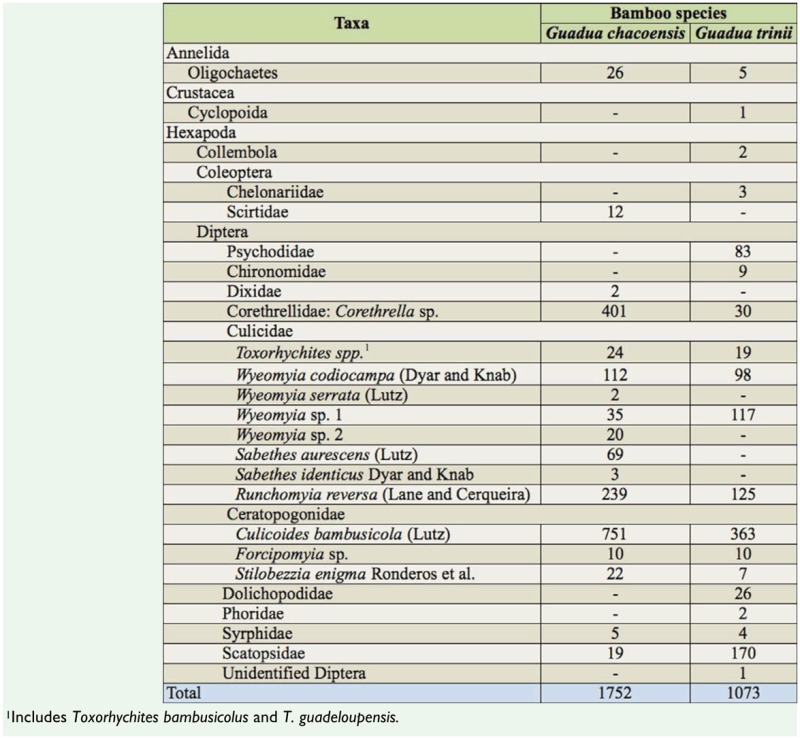
Taxonomic classification of the aquatic macro-invertebrates inhabiting two bamboos species from subtropical forest, Misiones Province, Argentina.

Taxa mainly were represented by immature insects. Diptera was the order best represented in the bamboo communities, with Culicidae as the dominant family, followed by Ceratopogonidae. Richness of Culicidae species was higher in *G. chacoensis* (n = 9) than in *G. trinii* (n = 5), while no difference was observed between bamboos with respect to Ceratopogonidae. Coleoptera were occasionally present in the communities, being represented mainly by species of semi-aquatic habitats, such as scirtids and chelonariids.

Eleven taxa were present in both species of bamboo, and 6 were unique to *G. chacoensis* and 8 to *G. trinii* (Table 3). Regarding the taxa unique to each bamboo, *G. chacoensis* hosted a greater number of species of Culicidae while *G. trinii* hosted more species belonging to other dipterous families.

The 2 *Guadua* phytotelmata were significantly different (*t* = -35.46; df = 48; *p* < .001) in terms of the diversity and evenness of macroinvertebrates occurring in them. The diversity was greater in *G. trinii* (*H*' = 2.03; *E* = 0.69) than in *G. chacoensis* (*H*' = 1.74; *E* = 0.61). The Berger-Parker index of dominance (*d*) was not significantly different (*t* = -1; df = 78; *p* = 0.33) between *G. trinii* (*d* = 0.60 ± 0.20) and *G. chacoensis* (*d* = 0.56 ± 0.17). Morisita-Horn index of similarity was high (*C_MH_* = 0.80) because the internodes of both *Guadua* species shared most of the taxa.

#### Species association

Four values of the species association coefficient (C_8_) for taxa inhabiting *G. chacoensis* were significant between species of mosquitoes, and 5 between mosquitoes and other dipterans. *Toxorhynchites* spp. (Diptera: Culcidae) was negatively associated with *Sabethes aurescens* (Lutz) (C_8_ = -1, *p* < 0.05) and *Wyeomyia codiocampa* Dyar and Knab (C_8_ = -0.43, *p* < 0.01), while *S. aurescens* and *W. codiocampa* showed maximum positive association (C_8_ = 1, *p* < 0.05). On the other hand, both species of *Sabethes* (*S. identicus* Dyar and Knab and *S. aurescens*) were not associated (C_8_ = 0, *p* <0.05). Cg significant values for associations between other dipterans and species of culicids were: Syrphidae showed a slight positive association with *Toxorhynchites* spp. (C_8_ = 0.10, *p* < 0.05) but was not associated with *Wyeomyia serrata* (Lutz) (C_8_ = 0, *p* < 0.05). Cg values for species of Ceratopogonidae related to *Wyeomyia* sp. 1 and *Runchomyia reversa* (Lane and Cerqueira) Were low and positive (C_8_ *S. enigma-Wyomyia* sp. 1 = 0.32, *p* < 0.01; C_8 *C. bambusicola-R. reversa*_ = 0.43, *p* <0.05); in contrast, the association between Dixidae and *R. reversa* was high and negative (C_8_ = -1, *p* < 0.05).

Two C_8_ values were significant among taxa in *G. trinii*. Ceratopogonidae, *Stilobezzia enigma* Ronderos, Spinelli, and Borkent and *Forcipomyia* sp. (C_8_ = 0.60, *p* < 0.001), and Scatopsidae and Dolichopodidae (C_8_ = 0.50, *p* < 0.01) showed significant positive associations. In *G. trinii*, no associations were observed between species of Culicidae in contrast to *G. chacoensis*.

#### Abiotic parameters effects on aquatic communities

All morphological variables were used for the analysis, as no collinearity was detected (*VIF_G. chacoensis_* < 1.86; *VIF_G. trinii_* < 1.96). Durbin-Watson test for independence of residuals detected no autocorrelation between variables for both bamboos (*G. chacoensis*: *D*_N° indiv._ = 2.3; *D*_N° Taxa_ = 2.2; *G. trinii*: *D*_N° indiv._ = 2.3; *D*_N° Taxa_ = 1.9). The multiple regression model for *G. chacoensis* explained 42% of abundance variation of macroinvertebrates and was significant (F = 3.59; df = 6, 30; *p* < 0.01), but, the model was not significant for the number of taxa (*r*^2^ = 0.25; F = 1.67; df = 6, 30; *p* = 0.16). By contrast, for *G. trinii*, the number of individuals and the number of taxa were significant (F_N° indiv._ = 3.62; df = 6, 34; *p* < 0.01; F_N°Taxa_ = 5.91; df = 6, 34; *p* < 0.001), and the model explained 39 and 51% respectively of the variation of these variables.

**Table 4.**
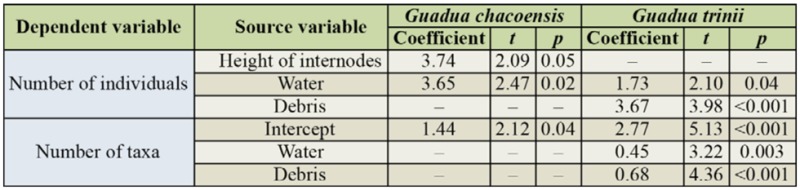
Coefficient and Student's t-test results (significant differences) from the multiple regression analysis from transformed data between macroinvertebrate individuals and number of taxa against abiotic variables of two bamboo phytotelmata.

**Table 5.**
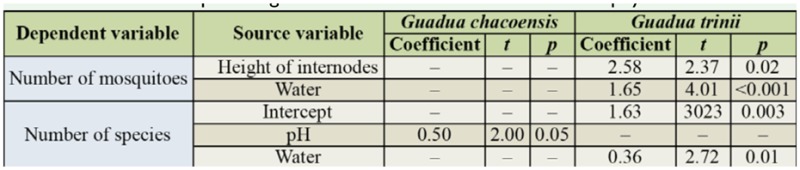
Coefficient and Student's t-test result (significant differences) from the multiple regression analysis from transformed data between number of mosquitoes and number of species against abiotic variables of two bamboo phytotelmata.

The number of individuals in the community that lived in the internodes of *G. chacoensis* was positively correlated with the volume of water and the height of the internodes. For *G. trinii*, the volume of water and the detritus correlated positively with both the number of individuals and the number of taxa (Table 4).

The independent variables regressed against species of mosquitoes were not autocorrelated for either species of bamboo (*G. chacoensis*:*D*_N° mosq._ = 2.2; *D*_N° species_ = 2, *G. trinii*: *D*_N° mosq._ = 1.8; *D*_N° species_ = 1.8). The multiple regression model for *G. chacoensis* was significant (F = 2.90; df = 6, 30; *p* *<* 0.05) and explained 37% of the variation of abundance and number of species. For *G. trinii*, it explained 42% of the variation of the abundance of mosquitoes and 26% of the variation of the number of species, but was only significant for abundance (F_N° mosq_. = 4.1; df = 6, 34; *p* < 0.01; F_N° species_ = 2.90; df = 6, 34; *p* = 0.1).

The abundance of mosquitoes and the number of species that inhabited internodes of *G. chacoensis* was correlated with pH, while for abundance of mosquitoes in *G. trinii* the correlated variables were the water volume and the height of the internodes, and for the number of species the water volume (Table 5).

As previous studies on bamboo internodes (e.g., Kurihara, 1959, 1983) showed that the occurrence of the species of mosquitoes is related to the pH of the standing water in bamboo internodes, the correlation coefficients were calculated for the number of mosquitoes per genera that made up the community of both species of *Guadua* phytotelmata. Although all species of mosquitoes were found in internodes with pH ranging from strongly acidic to alkaline, no significant correlations were observed in any of them (*G. chacoensis*: *r*_*s, Toxorhynchites spp*._ = 0.13, *p* = 0.15, n = 23, *r_s, Wyeomyia spp._* = -0.15, *p* = 0.44, n = 27; *r*_*s*, *R*. reversa_= 0.17, *p* = 0.39, n = 26; *r*_*s, Sabethes* spp_. = -0.32, *p* = 0.75, n = 4; *G. trinii*: *r_S, Wyeomyia spp._* = 0.13, *p* = 0.49, n = 31; *r_S, R. reversa_* = -0.10, *p* = 0.66, n = 22). All species were predominately in internodes with an acidic pH.

#### Predators

Seven predators were identified from both species of bamboo: *Toxorhynchites bambusicolus* (Lutz and Neiva), *T. guadeloupensis* (Dyar and Knab), *R. reversa*, *S. aurescens*, *S. identicus*, *Culicoides bambusicola* (Lutz), and *Corethrella* sp. Species of *Toxorhynchites* are the biggest predators in the internodes, *R. reversa*, *S. aurescens*, *S. identicus* are medium and potential facultative predators (as are other species of *Sabethes*) (Lozovei and Luz 1976, Machado-Allison 1986), and *C. bambusicola* and the Corethrellidae are the smallest.

**Figure 2.**
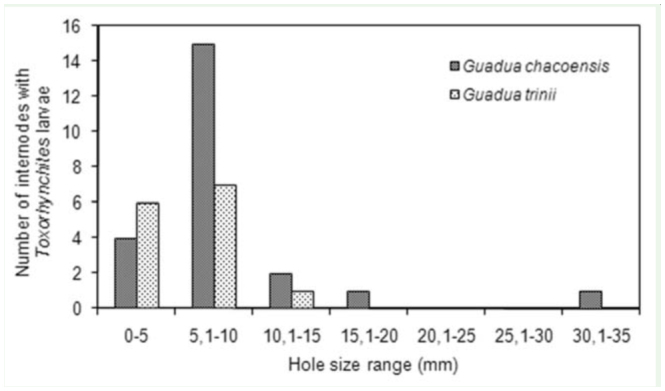
Number of internodes of *Guadua chacoensis* and *G. trinii* inhabited by *Toxorhynchites* larvae segregated into hole size categories. High quality figures are available online.

Because the species of *Toxorhynchites* are the largest predators in both *G. chacoensis* and *G. trinii*, it was of interest to compare the occupancy of the internodes with different size entry holes in these species. Respectively, 59 and 46.3% of internodes of *G. chacoensis* and *G. trinii* were inhabited by larvae of *Toxorhynchites* spp. All internodes contained 1 larva, except 1 *G. chacoensis* contained two. Most internodes with *Toxorhynchites* had entry holes of less than 10 mm. There were no larvae of *Toxorhynchites* in internodes with entry holes of 20–30 mm, and only 1 larva was collected from internodes of *G. chacoensis* with a hole greater than 30 mm (Figure 2). The smallest inlet size in an internode where a *Toxorhynchites* larva was found was 3 mm in a *G. trinii*. However the proportion of internodes with entry holes less than 10 mm containing *Toxorhynchites* larvae was not significantly different from internodes with entry holes greater than 10 mm for both *G. chacoensis* (*G _adj_*. = 0.02, *p* > 0.50) and *G. trinii* (*G _adj_*. = 0.42, *p* > 0.50).

To determine whether prey abundance was affected by the presence of the predator *Toxorhynchites* in each internode of both bamboo species, the numbers of individual prey collected from these two habitats were compared. Mann-Whitney rank tests showed no significant differences between the number of prey in internodes with or without *Toxorhynchites* larvae (*U _G. chacoensis_* = 302, n _Min._ = 16, n _Max._ = 23, *p* = 0.62; *U _G. trinii_* = 421, n _Min._ = 19, n_Max._ = 22, *p* = 0.57).

### Discussion

#### Bamboo internodes as micro-habitat

**Figure 3.**
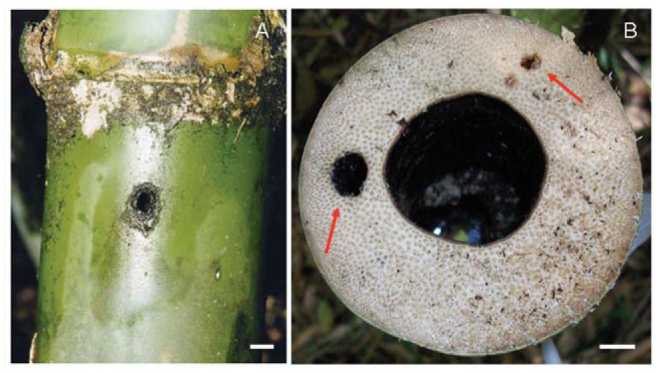
*Guadua chacoensis*: A- View of an entry hole to an internode, B- Cross-section showing the wall thickness of an internode and galleries (indicated by arrows) formed by a boring beetle. Scaling = I cm. High quality figures are available online.

Macroinvertebrates access internodes through holes caused by the boring activities of animals such as leaf beetles, long-horned beetles, caterpillars, or woodpeckers in the walls of culms (Kovac and Streit 1996), producing holes of different shapes and sizes. The inlet holes of *G. chacoensis* were smooth, circular drillings (Figure 3a), so they could have been caused by a boring beetle, as the wall of the stem was extremely hard and thick (∼ 3 cm). Moreover, internal galleries were observed when bamboos were cut (Figure 3b). The same circular inlet holes, but of smaller diameter, were observed in *G. trinii*, although they were accompanied by grooves and irregular holes. This heterogeneity of shapes and sizes could be because the stem of *G. trinii* was much thinner (∼ 5 mm) than the stem of *G. chacoensis*, and can be attacked by several species of arthropods.

Factors with significant loading from principal component analyses included internal diameter of internodes and the height of the entry hole, contributing to internode capacity to retain more water and detritus. The water retained in bamboo internodes comes from the plant exudates (Louton et al. 1996) and from runoff during heavy rains (Kovac and Streit 1996), leading to large variations in volume of water held by internodes. Louton et al. (1996) observed that the volume of water in *G. weberbaueri* had a range from 1–325 mL, and noted stains below the lateral opening which were attributed to frequent overflows, apparently, when internodes are filled with water above the level of the openings. Similar observations were made in *G. chacoensis* and *G. trinii*, which showed differences not only in the volumes of water from the internodes of both *Guadua*, but also between the internodes of each species.

During the present study, spill stains were also noted, as observed by Louton et al. (1996), although the volume of water never exceeded 60% of the capacity of the internodes.

The origin of food resources in the internodes came from the runoff produced when heavy rainwater seeped through the bamboo, and from the faeces, exuviae, and corpses of macroinvertebrates that inhabit them (Personal observation). Among the internodes of *G. chacoensis*, there was greater variation in the amount of debris retained than observed for *G. trinii*. The higher variation in *G. chacoensis* could be related to higher entry holes (as shown in the principal component analysis diagram), allowing the entry of terrestrial invertebrates that use the upper portion of the internodes as a refuge (Louton et al. 1996). These invertebrates would contribute to the debris from defecation and/or food waste (Mogi and Suzuki 1983). Lozovei (2001) mentioned that the water in live internodes is acidic (3.5) or neutral (7.1). Unlike the results of that author, the pH of the water retained in the internodes of both species of *Guadua* was predominantly acidic (pH: minimum 2.7, mean: ∼ 5), reaching strongly alkaline values (8.3) in *G. chacoensis* and slightly alkaline (7.7) in *G. trinii*.

#### Fauna communities

The aquatic communities that inhabit bamboo are mainly composed of immatures of several dipterans (Louton et al. 1996), the Culicidae being the most diverse and most studied family. In the aquatic communities of South American bamboos, the mosquitoes are the principal inhabitants, within which Sabethini is the most diverse and dominant tribe. Approximately 40 species of Culicidae were reported from bamboo phytotelmata, of which 14 and 12 identified and unidentified species, respectively, have been found in *Guadua* phytotelmata (Louton et al. 1996; Lozovei 1998, 2001; Campos et al. 2011). In a recent study from subtropical Argentina, 9 identified species collected from *Guadua* were reported (Campos et al. 2011). In the present study, the same species were recorded, except *Trichoprosopon pallidiventer* (Lutz) and *Wyeomyia sabathea* Lane and Cerqueira. From the species occurring in *Guadua* phytotelmata, only *Tx. bambusicola* and *Tr. pallidiventer* have been recorded from containers other than bamboo phytotelmata (Campos et al. 2011) in the study area. Other species of mosquitoes (Table 3) can be considered as specialists of bamboo phytotelmata because they were not found in other micro-habitats. It is worth noting that no species of the genus *Culex* was found in the internodes of bamboos in the present study, as opposed to the abundance found by other authors (Louton et al. 1996; Lozovei 1998, 2001) who studied the fauna inhabiting *Guadua* bamboo in Brazil and Peru. Of the identified species of Cerato-pogonidae occurring in bamboo phytotelmata, *C. bambusicola*, (Ronderos and Spinelli 2000) and *S. enigma* (Ronderos et al. 2012) are inhabitants with no record from other habitats; by contrast, *Palpomyia guarani* was collected not only from bamboos, but also from treeholes (Ronderos et al. 2004).

Other dipterans present in Neotropical phytotelmata are the Corethrellidae, with 19 species restricted to treeholes and bamboo internodes (Borkent 2008). However, in the present study, only an unidentified species of *Corethella*, which was the second most abundant Dipteran, was recorded in both species of *Guadua* phytotelmata.

#### Species association

In a study carried out in Perú, 2.7 taxa per internode of *G. weberbaueri* were recorded (Louton et al. 1996). By contrast, the results of the present study showed a mean of 4.6 and 4 taxa per internode of *G. chacoensis* and *G. trinii*, respectively. The present study showed high species associations, both positive and negative. Negative associations with predators were possible due to asynchronies in occurrence with other macroinvertebrates in the internodes. While the observed positive association occurred between predators and syrphids, this would suggest that they may not be a prey chosen by the predator, possibly due to their larger size or because the syrphids possess some behavior for escaping from predators. The filter feeding *Sabethes aurescens* and *W. codiocampa* were strongly associated, suggesting that competition for resources is low. By contrast, both species of *Sabethes* (*S. aurescens* and *S. identicus*) were not associated.

#### Abiotic parameters effects on aquatic communities

The abundance of macroinvertebrates in the water-filled *Guadua* was attributed to the size of the perforation on the side of the internodes rather than to the volume of water or the height of the opening above ground, while the number of taxa was not associated with any abiotic parameter of internodes (Louton et al. 1996). The results showed that the volume of water and debris, and to a lesser extent the height of the opening above ground, are parameters that strongly influence the aquatic community. The size of the entry hole to the internodes could function as a mechanism for selecting the size of the individuals rather than their abundance. Louton et al. (1996) analyzed the community of mosquitoes in particular, and found that both the number of individuals and the number of species were significantly correlated with the volume of water. Conversely, Lozovei (2001) noted that the number of mosquitoes depends on the volume of water retained within the internodes. The results of both *Guadua* studied here are ambiguous. The abundance and richness of species of mosquitoes that developed in the internodes of *G. trinii* were correlated with the volume of water, while the mosquitoes that developed in *G. chacoensis* were correlated to pH but not to the volume of water.

The succession of dipteran larvae in the bamboo is likely to occur as a result of successive changes in pH, and it is noted, for example, that the mosquito *Aedes flavopictus* colonizes internodes when the pH is acidic (pH ∼ 6.2), while *Armigeres subalbatus* colonizes them when the medium becomes slightly alkaline (pH ∼ 7.7) (Kurihara 1983). No mosquito larvae were found when the pH was less than 5.5 (Kurihara 1959). The results found in the present study, unlike those of Kurihara (1983, 1959), showed that the species of mosquitoes inhabiting the internodes of *Guadua* bamboo (all Sabethini except *Toxorhynchites* spp.) were related preferably to acidic water, tolerating pH values well below 5.5 and being less frequent in alkaline water. Similar results were found by Lozovei (2001) in a study on mosquitoes that inhabit *Merostachys* bamboo internodes in Brazil.

#### Predators

Immatures of *Toxorhynchites*, *Corethrella* sp., and *C. bambusicola* were the main predators in the aquatic communities of both *Guadua*. Other members of the community, as some genera of mosquitoes of the Sabethini tribe, are suspected to be predators, among them the species of *Runchomyia* and *Sabethes*. All these predators, except *Toxorhynchites* larvae, showed aggregated inter-bamboo distribution. On the contrary, the predator *Toxorhynchites*, whose cannibalism has been widely documented (e.g., Lounibos et al. 1996; Campos and Lounibos 2000a, b), usually occurred alone in each internode. A similar observation was reported by Kovac and Streit (1996) from bamboo internodes in Malaysia.

Predation by Corethrellidae has been well-documented by Griswold and Lounibos (2006), who studied the effects of predation by *Corethrella appendiculata* in treeholes. These authors concluded that *C. appendiculata* larvae change species composition by preferentially consuming a prey type, while *Toxorhynchites* larvae by reducing prey density, regardless of the species present in the community. In another study, carried out on predatory habits of dipteran larvae inhabiting *'Nepenthes* pitchers, it was observed that *Corethrella calathicola* larvae attack younger culicids and immature ceratopogonids, and when prey are scarce they may develop a cannibalistic behavior (Mogi and Chan 1996). Predation by *Corethrella* was not examined here, but due to the aforementioned studies, it is assumed it can be a significant predator dueto its abundance, mainly in those internodes that are not occupied by *Toxorhynchites* larvae.

It is known that larval feeding habits of New Word Sabethini are facultative predators or filter feeding (Judd 1996). For example, it was observed in laboratory that the *Isostomyia paranensis* larvae (sister groups of *Runchomyia*) prey on other larvae of mosquitoes, grasping the body and sucking the soft tissues without taking the skin (Campos and Zavortink 2010), and *Runchomyia frontosa* occasionally preys on inhabitants of phytotelmata (Zavortink 1986). Some species of *Sabethes* are known as facultative predators, preying on culicids, ceratopogonids and other larvae of aquatic dipterans (Lozovei and Luz 1976; Machado-Allison 1976). Predation by *R. reversa*, *S. aurescens*, and *S. identicus* has not been documented. However, due to the fact that their mouthparts are similar to the ones of other species of the same genus (Harbach and Peyton 1993), they could be potential facultative predators in the aquatic communities of *Guadua*.

#### Conclusions

On the basis of the results of this article, it is concluded that: (1) *G. chacoensis* provided more aquatic habitats for the macroinvertebrate community than *G. trinii*; (2) the entry hole determined directly (by limiting the size of debris) and indirectly (by limiting the size of visitors that provide faeces and corpses to the water) the resource availability; (3) the communities differed between *Guadua* species, but a large number of macroinvertebrates used both phytotelmata; (4) the richness of species found in both species showed that these phytotelmata were mainly colonized by species of Culicidae and Ceratopogonidae; and (5) the largest predators, which tended to occur singly, were mosquito larvae of the genus *Toxorhynchites*.

## Abbreviations:

C8,: Hulbert's coefficient of association;
**CV**,: coefficient of variation;
**D**,: Durbin-Watson statistic;
**d**,: Berger-Parker index of dominance;
*de*,: depth;
E,: evenness;
*G_adj_*,: G test;
*H*',: Shannon-Wiener index of diversity;
*I*,: length;
***r^2^***,: Pearson's coefficient of correlation;
*r*s,: Spearman's coefficient of correlation;
*VIF*,: variance inflation factor.
